# (*E*)-*N*-Benzyl­idene-4*H*-1,2,4-triazol-4-amine

**DOI:** 10.1107/S1600536810003946

**Published:** 2010-02-06

**Authors:** M. Thenmozhi, T. Kavitha, B. Palakshi Reddy, V. Vijayakumar, M. N. Ponnuswamy

**Affiliations:** a Centre of Advanced Study in Crystallography and Biophysics, University of Madras, Guindy Campus, Chennai 600 025, India; b Organic Chemistry Division, School of Advanced Sciences, VIT University, Vellore 632 014, India

## Abstract

The title compound, C_9_H_8_N_4_, crystallizes with three independent mol­ecules (*A*, *B* and *C*) per asymmetric unit. The independent mol­ecules differ slightly in their conformations, the dihedral angles between the triazole and phenyl rings in mol­ecules *A*, *B* and *C* being 4.8 (2), 9.7 (2) and 7.2 (2)°, respectively. In the crystal, the independent mol­ecules are linked into a trimer by C—H⋯N hydrogen bonds.

## Related literature

For the biological activity of triazole derivatives, see: Demirbas *et al.* (2002 ▶); Foroumadi *et al.* (2003 ▶); He *et al.* (2006 ▶); Kritsanida *et al.* (2002 ▶); Manfredini *et al.* (2000 ▶). For C—N and C=N bond-length data, see: Jin *et al.* (2004 ▶); Xiang *et al.* (2004 ▶). For graph-set analysis, see: Bernstein *et al.* (1995 ▶).
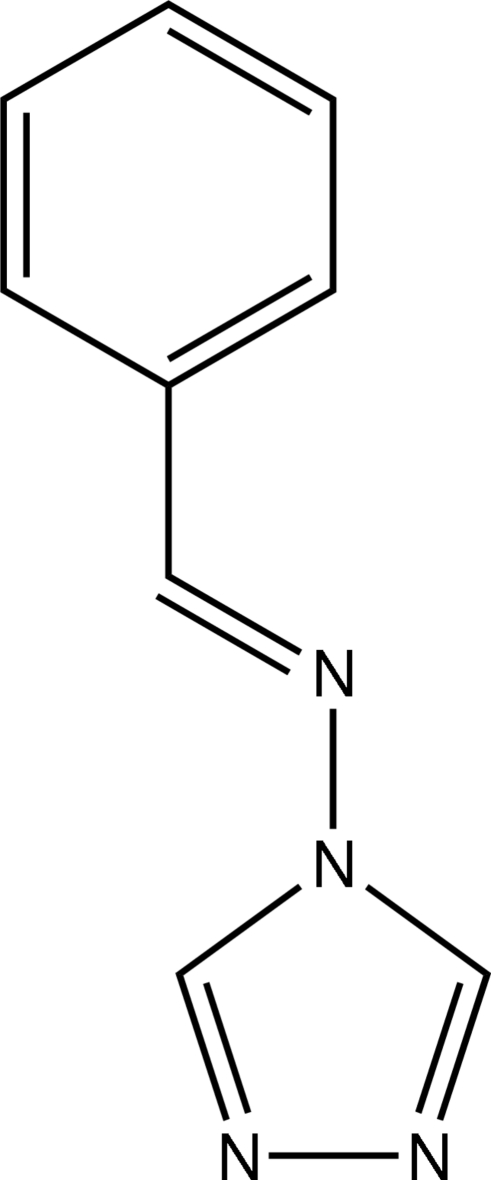


## Experimental

### 

#### Crystal data

C_9_H_8_N_4_
*M* *_r_* = 172.19
Monoclinic, 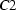

*a* = 33.0059 (11) Å
*b* = 4.0639 (1) Å
*c* = 20.6535 (7) Å
β = 111.067 (2)°
*V* = 2585.14 (14) Å^3^
*Z* = 12
Mo *K*α radiation
μ = 0.09 mm^−1^
*T* = 293 K
0.20 × 0.15 × 0.12 mm

#### Data collection

Bruker Kappa APEXII area-detector diffractometer
Absorption correction: multi-scan (*SADABS*; Sheldrick, 2001 ▶) *T*_min_ = 0.983, *T*_max_ = 0.990
14802 measured reflections
3418 independent reflections
2582 reflections with *I* > 2σ(*I*)
*R*_int_ = 0.032

#### Refinement

*R*[*F* ^2^ > 2σ(*F* ^2^)] = 0.039
*wR*(*F* ^2^) = 0.133
*S* = 0.99
3418 reflections
352 parameters
1 restraint
H-atom parameters constrained
Δρ_max_ = 0.13 e Å^−3^
Δρ_min_ = −0.16 e Å^−3^

### 

Data collection: *APEX2* (Bruker, 2004 ▶); cell refinement: *SAINT* (Bruker, 2004 ▶); data reduction: *SAINT*; program(s) used to solve structure: *SHELXS97* (Sheldrick, 2008 ▶); program(s) used to refine structure: *SHELXL97* (Sheldrick, 2008 ▶); molecular graphics: *PLATON* (Spek, 2009 ▶) and *ORTEP-3* (Farrugia, 1997 ▶); software used to prepare material for publication: *SHELXL97* and *PLATON* (Spek, 2009 ▶).

## Supplementary Material

Crystal structure: contains datablocks global, I. DOI: 10.1107/S1600536810003946/ci5006sup1.cif

Structure factors: contains datablocks I. DOI: 10.1107/S1600536810003946/ci5006Isup2.hkl

Additional supplementary materials:  crystallographic information; 3D view; checkCIF report

## Figures and Tables

**Table 1:** Hydrogen-bond geometry (Å, °)

*D*—H⋯*A*	*D*—H	H⋯*A*	*D*⋯*A*	*D*—H⋯*A*
C3*C*—H3*C*⋯N1*B*	0.93	2.54	3.414 (4)	157
C7*C*—H7*C*⋯N1*B*	0.93	2.49	3.405 (3)	167
C3*A*—H3*A*⋯N2*C*^i^	0.93	2.47	3.387 (4)	169
C7*A*—H7*A*⋯N2*C*^i^	0.93	2.58	3.503 (3)	174
C5*B*—H5*B*⋯N2*A*^ii^	0.93	2.47	3.371 (4)	162

Symmetry codes: (i) 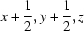; (ii) 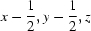.

**Fig. 1.**
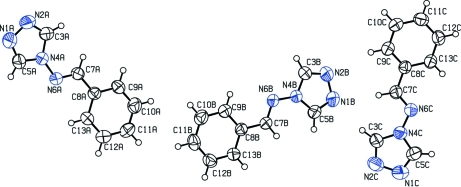
The asymmetric unit of the title compound. Displacement ellipsoids are drawn at the 50% probability level.

**Fig. 2.**
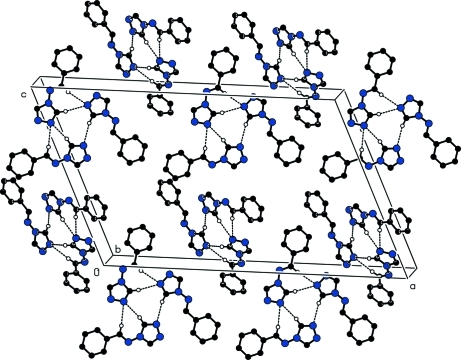
The crystal packing of the title compound, showing the formation of hydrogen-bonded (dashed lines) trimers.

## supplementary crystallographic information

### Comment

1,2,4-Triazole is a basic aromatic ring and possesses good coordination ability due to the presence of nitrogen atoms. The 1,2,4-triazole derivatives are used to build polymetallic complexes (He *et al.*, 2006). Compounds derived from triazole possess antimicrobial, analgesic, anti-inflammatory, local anesthetic, antineoplastic and antimalarial properties (Foroumadi *et al.*, 2003). Some triazole Schiff bases also exhibit antiproliferative and anticancer activities (Manfredini *et al.*, 2000). Due to their significant biological applications, triazoles have gained much attention in bioinorganic and metal-based drug discovery (Demirbas *et al.*, 2002; Kritsanida *et al.*, 2002).

There are three crystallographically independent molecules (A, B and C) in the asymmetric unit (Fig. 1). The 1,2,4-triazole ring (N1/N2/C3/N4/C5) is planar and attached to the phenyl ring (C8-C13) through the C═N bond. The dihedral angles between triazole and phenyl rings are 4.8 (2)°, 9.7 (2)° and 7.2 (2)° for molecules A, B and C, respectively. The C—N bond lengths in the triazole ring of all molecules lie in the range of 1.260 (3)–1.349 (4) Å. These are longer than a typical double C═N bond [1.269 (2) Å] (Xiang *et al.*, 2004), but shorter than a C—N single bond [1.443 (4) Å] (Jin *et al.*, 2004), indicating the possibility of electron delocalization.

The crystal packing (Fig 2) shows that the indepentent molecules exist as C—H···N hydrogen-bonded trimers.

### Experimental

A mixture of benzaldehyde (10 mmol) and 4-amino-4H-1,2,4-triazole (10 mmol) in ethanol was refluxed on a steam-bath for 30 min. The colour of the solution changed to reddish-orange and was kept under ice-cold conditions to obtain a white solid product. Single crystals were formed in the mother liquor after five days.

### Refinement

H atoms were positioned geometrically (C-H = 0.93 Å) and allowed to ride on their parent atoms, with *U*_iso_(H) = 1.2 *U*_eq_(C).

### Crystal data

**Table:**

C_9_H_8_N_4_	*F*(000) = 1080
*M**_r_* = 172.19	*D*_x_ = 1.327 Mg m^−^^3^
Monoclinic, *C*2	Mo *K*α radiation, λ = 0.71073 Å
Hall symbol: C 2y	Cell parameters from 14802 reflections
*a* = 33.0059 (11) Å	θ = 2.1–27.6°
*b* = 4.0639 (1) Å	µ = 0.09 mm^−^^1^
*c* = 20.6535 (7) Å	*T* = 293 K
β = 111.067 (2)°	Block, colourless
*V* = 2585.14 (14) Å^3^	0.20 × 0.15 × 0.12 mm
*Z* = 12	

### Data collection

**Table:**

Bruker Kappa APEXII area-detector diffractometer	3418 independent reflections
Radiation source: fine-focus sealed tube	2582 reflections with *I* > 2σ(*I*)
graphite	*R*_int_ = 0.032
ω and φ scans	θ_max_ = 27.6°, θ_min_ = 2.1°
Absorption correction: multi-scan (*SADABS*; Sheldrick, 2001)	*h* = −42→42
*T*_min_ = 0.983, *T*_max_ = 0.990	*k* = −4→5
14802 measured reflections	*l* = −26→26

### Refinement

**Table:**

Refinement on *F*^2^	Primary atom site location: structure-invariant direct methods
Least-squares matrix: full	Secondary atom site location: difference Fourier map
*R*[*F*^2^ > 2σ(*F*^2^)] = 0.039	Hydrogen site location: inferred from neighbouring sites
*wR*(*F*^2^) = 0.133	H-atom parameters constrained
*S* = 0.99	*w* = 1/[σ^2^(*F*_o_^2^) + (0.0936*P*)^2^] where *P* = (*F*_o_^2^ + 2*F*_c_^2^)/3
3418 reflections	(Δ/σ)_max_ = 0.005
352 parameters	Δρ_max_ = 0.13 e Å^−^^3^
1 restraint	Δρ_min_ = −0.16 e Å^−^^3^

### Special details

**Table:**

Geometry. All esds (except the esd in the dihedral angle between two l.s. planes) are estimated using the full covariance matrix. The cell esds are taken into account individually in the estimation of esds in distances, angles and torsion angles; correlations between esds in cell parameters are only used when they are defined by crystal symmetry. An approximate (isotropic) treatment of cell esds is used for estimating esds involving l.s. planes.
Refinement. Refinement of F^2^ against ALL reflections. The weighted R-factor wR and goodness of fit S are based on F^2^, conventional R-factors R are based on F, with F set to zero for negative F^2^. The threshold expression of F^2^ > 2sigma(F^2^) is used only for calculating R-factors(gt) etc. and is not relevant to the choice of reflections for refinement. R-factors based on F^2^ are statistically about twice as large as those based on F, and R- factors based on ALL data will be even larger.

### Fractional atomic coordinates and isotropic or equivalent isotropic displacement parameters (Å^2^)

**Table:**

	*x*	*y*	*z*	*U*_iso_*/*U*_eq_	
N1A	1.09649 (8)	0.8835 (8)	0.61511 (13)	0.0763 (7)	
N2A	1.10029 (7)	0.8972 (8)	0.68405 (12)	0.0759 (7)	
C3A	1.06740 (8)	0.7385 (9)	0.68915 (13)	0.0669 (8)	
H3A	1.0622	0.7106	0.7302	0.080*	
N4A	1.04188 (6)	0.6199 (6)	0.62682 (10)	0.0531 (5)	
C5A	1.06160 (9)	0.7183 (9)	0.58349 (14)	0.0683 (8)	
H5A	1.0513	0.6726	0.5362	0.082*	
N6A	1.00305 (6)	0.4455 (6)	0.60450 (10)	0.0534 (5)	
C7A	0.98725 (7)	0.3748 (7)	0.64995 (11)	0.0499 (5)	
H7A	1.0022	0.4344	0.6959	0.060*	
C8A	0.94615 (7)	0.2018 (6)	0.63197 (11)	0.0480 (5)	
C9A	0.92932 (8)	0.1395 (8)	0.68311 (12)	0.0566 (6)	
H9A	0.9448	0.2018	0.7287	0.068*	
C10A	0.88964 (9)	−0.0149 (8)	0.66666 (14)	0.0678 (8)	
H10A	0.8782	−0.0523	0.7011	0.081*	
C11A	0.86711 (8)	−0.1130 (8)	0.60023 (14)	0.0643 (7)	
H11A	0.8404	−0.2169	0.5895	0.077*	
C12A	0.88395 (8)	−0.0582 (8)	0.54891 (13)	0.0601 (6)	
H12A	0.8689	−0.1293	0.5038	0.072*	
C13A	0.92287 (8)	0.1007 (7)	0.56441 (12)	0.0533 (6)	
H13A	0.9338	0.1415	0.5295	0.064*	
N1B	0.65846 (7)	0.6473 (9)	0.90074 (12)	0.0818 (9)	
N2B	0.69659 (7)	0.7618 (10)	0.94996 (11)	0.0836 (9)	
C3B	0.71899 (8)	0.8843 (11)	0.91654 (12)	0.0724 (9)	
H3B	0.7461	0.9809	0.9374	0.087*	
N4B	0.69836 (6)	0.8560 (7)	0.84744 (9)	0.0559 (6)	
C5B	0.66061 (8)	0.7056 (10)	0.84063 (13)	0.0710 (9)	
H5B	0.6391	0.6515	0.7985	0.085*	
N6B	0.71639 (6)	0.9630 (7)	0.79949 (9)	0.0548 (5)	
C7B	0.69559 (7)	0.8917 (7)	0.73663 (11)	0.0500 (6)	
H7B	0.6691	0.7835	0.7244	0.060*	
C8B	0.71297 (7)	0.9785 (7)	0.68307 (11)	0.0474 (5)	
C9B	0.75212 (8)	1.1431 (8)	0.69885 (13)	0.0561 (6)	
H9B	0.7673	1.2118	0.7441	0.067*	
C10B	0.76865 (8)	1.2052 (8)	0.64756 (14)	0.0630 (7)	
H10B	0.7951	1.3128	0.6583	0.076*	
C11B	0.74595 (9)	1.1078 (8)	0.58065 (13)	0.0636 (7)	
H11B	0.7570	1.1516	0.5460	0.076*	
C12B	0.70704 (8)	0.9466 (8)	0.56432 (12)	0.0601 (7)	
H12B	0.6919	0.8808	0.5189	0.072*	
C13B	0.69040 (7)	0.8821 (7)	0.61552 (11)	0.0535 (6)	
H13B	0.6640	0.7737	0.6045	0.064*	
N1C	0.49788 (8)	0.0614 (8)	0.83722 (13)	0.0784 (7)	
N2C	0.53653 (9)	0.0797 (9)	0.82568 (12)	0.0807 (8)	
C3C	0.56350 (9)	0.2426 (10)	0.87673 (13)	0.0710 (8)	
H3C	0.5920	0.2902	0.8817	0.085*	
N4C	0.54458 (6)	0.3340 (6)	0.92178 (10)	0.0537 (5)	
C5C	0.50405 (9)	0.2134 (9)	0.89449 (15)	0.0684 (8)	
H5C	0.4831	0.2370	0.9145	0.082*	
N6C	0.55923 (6)	0.5052 (6)	0.98418 (10)	0.0539 (5)	
C7C	0.59724 (7)	0.6207 (7)	1.00274 (11)	0.0515 (6)	
H7C	0.6134	0.5897	0.9745	0.062*	
C8C	0.61623 (7)	0.8013 (6)	1.06753 (11)	0.0470 (5)	
C9C	0.65723 (7)	0.9332 (8)	1.08465 (12)	0.0563 (6)	
H9C	0.6722	0.9077	1.0545	0.068*	
C10C	0.67619 (8)	1.1023 (8)	1.14589 (13)	0.0597 (6)	
H10C	0.7038	1.1912	1.1570	0.072*	
C11C	0.65420 (8)	1.1391 (8)	1.19040 (13)	0.0610 (7)	
H11C	0.6671	1.2517	1.2320	0.073*	
C12C	0.61298 (8)	1.0098 (8)	1.17387 (13)	0.0600 (7)	
H12C	0.5981	1.0367	1.2041	0.072*	
C13C	0.59415 (7)	0.8429 (7)	1.11312 (12)	0.0539 (6)	
H13C	0.5664	0.7563	1.1021	0.065*	

### Atomic displacement parameters (Å^2^)

**Table:**

	*U*^11^	*U*^22^	*U*^33^	*U*^12^	*U*^13^	*U*^23^
N1A	0.0707 (14)	0.0899 (19)	0.0753 (15)	−0.0164 (15)	0.0347 (12)	−0.0053 (15)
N2A	0.0619 (13)	0.095 (2)	0.0652 (13)	−0.0217 (15)	0.0155 (11)	−0.0066 (14)
C3A	0.0572 (14)	0.087 (2)	0.0529 (13)	−0.0134 (16)	0.0155 (11)	−0.0027 (15)
N4A	0.0480 (10)	0.0627 (13)	0.0492 (10)	−0.0038 (10)	0.0182 (8)	−0.0027 (10)
C5A	0.0688 (16)	0.082 (2)	0.0625 (14)	−0.0130 (17)	0.0333 (13)	−0.0075 (15)
N6A	0.0476 (10)	0.0621 (13)	0.0494 (10)	−0.0056 (10)	0.0161 (8)	−0.0055 (10)
C7A	0.0499 (12)	0.0533 (14)	0.0449 (11)	0.0011 (11)	0.0152 (9)	−0.0030 (11)
C8A	0.0477 (12)	0.0482 (13)	0.0464 (11)	0.0034 (11)	0.0150 (9)	0.0016 (10)
C9A	0.0611 (14)	0.0658 (17)	0.0431 (11)	−0.0012 (13)	0.0191 (11)	0.0038 (12)
C10A	0.0656 (15)	0.078 (2)	0.0652 (15)	−0.0065 (16)	0.0305 (13)	0.0109 (16)
C11A	0.0503 (13)	0.0653 (17)	0.0736 (16)	−0.0079 (14)	0.0177 (12)	0.0058 (15)
C12A	0.0560 (14)	0.0621 (16)	0.0549 (13)	−0.0034 (13)	0.0109 (11)	−0.0022 (13)
C13A	0.0560 (13)	0.0593 (15)	0.0451 (11)	−0.0007 (12)	0.0190 (10)	−0.0021 (11)
N1B	0.0627 (13)	0.129 (3)	0.0611 (13)	−0.0159 (17)	0.0309 (11)	0.0037 (16)
N2B	0.0653 (14)	0.138 (3)	0.0515 (11)	−0.0044 (17)	0.0259 (11)	−0.0004 (16)
C3B	0.0552 (14)	0.117 (3)	0.0464 (12)	−0.0110 (18)	0.0202 (11)	−0.0078 (16)
N4B	0.0439 (10)	0.0822 (16)	0.0428 (9)	−0.0034 (11)	0.0171 (8)	−0.0026 (11)
C5B	0.0501 (13)	0.112 (3)	0.0515 (13)	−0.0165 (17)	0.0192 (11)	−0.0008 (16)
N6B	0.0440 (10)	0.0778 (16)	0.0455 (10)	−0.0073 (11)	0.0195 (8)	−0.0016 (10)
C7B	0.0395 (10)	0.0629 (15)	0.0464 (11)	−0.0009 (11)	0.0141 (9)	−0.0009 (11)
C8B	0.0421 (11)	0.0538 (14)	0.0459 (11)	0.0053 (10)	0.0153 (9)	0.0042 (11)
C9B	0.0500 (12)	0.0650 (17)	0.0522 (12)	−0.0025 (12)	0.0171 (10)	0.0005 (12)
C10B	0.0571 (14)	0.0670 (17)	0.0684 (15)	−0.0072 (14)	0.0267 (12)	0.0075 (14)
C11B	0.0725 (16)	0.0701 (18)	0.0584 (14)	0.0083 (15)	0.0359 (13)	0.0132 (14)
C12B	0.0618 (14)	0.0728 (18)	0.0436 (11)	0.0079 (15)	0.0163 (10)	0.0030 (13)
C13B	0.0472 (12)	0.0651 (16)	0.0459 (11)	0.0025 (12)	0.0138 (9)	−0.0010 (12)
N1C	0.0705 (15)	0.0883 (19)	0.0681 (14)	−0.0135 (14)	0.0149 (12)	−0.0114 (15)
N2C	0.0887 (17)	0.096 (2)	0.0575 (12)	−0.0238 (17)	0.0267 (12)	−0.0143 (15)
C3C	0.0706 (16)	0.093 (2)	0.0567 (14)	−0.0223 (17)	0.0310 (13)	−0.0140 (16)
N4C	0.0526 (11)	0.0584 (13)	0.0481 (10)	−0.0033 (10)	0.0159 (8)	0.0010 (10)
C5C	0.0530 (14)	0.077 (2)	0.0696 (16)	−0.0061 (15)	0.0148 (13)	−0.0102 (16)
N6C	0.0498 (10)	0.0622 (14)	0.0493 (10)	0.0010 (10)	0.0173 (8)	−0.0001 (10)
C7C	0.0500 (12)	0.0584 (15)	0.0483 (11)	0.0014 (12)	0.0203 (10)	0.0027 (12)
C8C	0.0430 (11)	0.0496 (13)	0.0475 (11)	0.0048 (10)	0.0152 (9)	0.0060 (10)
C9C	0.0470 (12)	0.0671 (16)	0.0562 (12)	−0.0006 (13)	0.0201 (10)	0.0000 (13)
C10C	0.0447 (12)	0.0682 (17)	0.0610 (14)	−0.0027 (13)	0.0128 (11)	−0.0013 (14)
C11C	0.0598 (14)	0.0662 (18)	0.0509 (12)	0.0048 (14)	0.0123 (11)	−0.0057 (13)
C12C	0.0619 (14)	0.0639 (17)	0.0604 (14)	0.0010 (13)	0.0296 (12)	−0.0061 (13)
C13C	0.0467 (12)	0.0607 (16)	0.0565 (13)	0.0009 (12)	0.0213 (10)	0.0012 (12)

### Geometric parameters (Å, °)

**Table:**

N1A—C5A	1.290 (4)	C8B—C13B	1.381 (3)
N1A—N2A	1.385 (3)	C8B—C9B	1.386 (3)
N2A—C3A	1.299 (4)	C9B—C10B	1.378 (3)
C3A—N4A	1.349 (3)	C9B—H9B	0.93
C3A—H3A	0.93	C10B—C11B	1.372 (4)
N4A—C5A	1.343 (3)	C10B—H10B	0.93
N4A—N6A	1.390 (3)	C11B—C12B	1.371 (4)
C5A—H5A	0.93	C11B—H11B	0.93
N6A—C7A	1.260 (3)	C12B—C13B	1.380 (3)
C7A—C8A	1.452 (3)	C12B—H12B	0.93
C7A—H7A	0.93	C13B—H13B	0.93
C8A—C9A	1.382 (3)	N1C—C5C	1.284 (4)
C8A—C13A	1.391 (3)	N1C—N2C	1.381 (3)
C9A—C10A	1.380 (4)	N2C—C3C	1.292 (4)
C9A—H9A	0.93	C3C—N4C	1.346 (3)
C10A—C11A	1.364 (4)	C3C—H3C	0.93
C10A—H10A	0.93	N4C—C5C	1.344 (3)
C11A—C12A	1.380 (4)	N4C—N6C	1.389 (3)
C11A—H11A	0.93	C5C—H5C	0.93
C12A—C13A	1.369 (4)	N6C—C7C	1.263 (3)
C12A—H12A	0.93	C7C—C8C	1.456 (3)
C13A—H13A	0.93	C7C—H7C	0.93
N1B—C5B	1.291 (3)	C8C—C9C	1.379 (3)
N1B—N2B	1.384 (4)	C8C—C13C	1.393 (3)
N2B—C3B	1.280 (4)	C9C—C10C	1.376 (4)
C3B—N4B	1.347 (3)	C9C—H9C	0.93
C3B—H3B	0.93	C10C—C11C	1.369 (3)
N4B—C5B	1.349 (3)	C10C—H10C	0.93
N4B—N6B	1.396 (2)	C11C—C12C	1.382 (4)
C5B—H5B	0.93	C11C—H11C	0.93
N6B—C7B	1.265 (3)	C12C—C13C	1.364 (4)
C7B—C8B	1.461 (3)	C12C—H12C	0.93
C7B—H7B	0.93	C13C—H13C	0.93
			
C5A—N1A—N2A	106.0 (2)	C9B—C8B—C7B	121.7 (2)
C3A—N2A—N1A	107.1 (2)	C10B—C9B—C8B	120.1 (2)
N2A—C3A—N4A	110.7 (2)	C10B—C9B—H9B	120.0
N2A—C3A—H3A	124.7	C8B—C9B—H9B	120.0
N4A—C3A—H3A	124.7	C11B—C10B—C9B	119.8 (2)
C5A—N4A—C3A	104.1 (2)	C11B—C10B—H10B	120.1
C5A—N4A—N6A	122.7 (2)	C9B—C10B—H10B	120.1
C3A—N4A—N6A	133.1 (2)	C12B—C11B—C10B	120.6 (2)
N1A—C5A—N4A	112.1 (2)	C12B—C11B—H11B	119.7
N1A—C5A—H5A	124.0	C10B—C11B—H11B	119.7
N4A—C5A—H5A	124.0	C11B—C12B—C13B	119.8 (2)
C7A—N6A—N4A	116.64 (18)	C11B—C12B—H12B	120.1
N6A—C7A—C8A	121.2 (2)	C13B—C12B—H12B	120.1
N6A—C7A—H7A	119.4	C8B—C13B—C12B	120.1 (2)
C8A—C7A—H7A	119.4	C8B—C13B—H13B	120.0
C9A—C8A—C13A	118.9 (2)	C12B—C13B—H13B	120.0
C9A—C8A—C7A	119.2 (2)	C5C—N1C—N2C	106.4 (2)
C13A—C8A—C7A	121.9 (2)	C3C—N2C—N1C	107.2 (2)
C10A—C9A—C8A	120.1 (2)	N2C—C3C—N4C	110.5 (2)
C10A—C9A—H9A	119.9	N2C—C3C—H3C	124.7
C8A—C9A—H9A	119.9	N4C—C3C—H3C	124.7
C11A—C10A—C9A	120.4 (2)	C5C—N4C—C3C	104.4 (2)
C11A—C10A—H10A	119.8	C5C—N4C—N6C	122.2 (2)
C9A—C10A—H10A	119.8	C3C—N4C—N6C	133.3 (2)
C10A—C11A—C12A	120.0 (2)	N1C—C5C—N4C	111.5 (2)
C10A—C11A—H11A	120.0	N1C—C5C—H5C	124.3
C12A—C11A—H11A	120.0	N4C—C5C—H5C	124.3
C13A—C12A—C11A	120.0 (2)	C7C—N6C—N4C	116.35 (18)
C13A—C12A—H12A	120.0	N6C—C7C—C8C	121.2 (2)
C11A—C12A—H12A	120.0	N6C—C7C—H7C	119.4
C12A—C13A—C8A	120.5 (2)	C8C—C7C—H7C	119.4
C12A—C13A—H13A	119.7	C9C—C8C—C13C	118.9 (2)
C8A—C13A—H13A	119.7	C9C—C8C—C7C	119.4 (2)
C5B—N1B—N2B	107.2 (2)	C13C—C8C—C7C	121.7 (2)
C3B—N2B—N1B	106.5 (2)	C10C—C9C—C8C	120.7 (2)
N2B—C3B—N4B	111.6 (3)	C10C—C9C—H9C	119.7
N2B—C3B—H3B	124.2	C8C—C9C—H9C	119.7
N4B—C3B—H3B	124.2	C11C—C10C—C9C	119.7 (2)
C3B—N4B—C5B	104.2 (2)	C11C—C10C—H10C	120.1
C3B—N4B—N6B	122.9 (2)	C9C—C10C—H10C	120.1
C5B—N4B—N6B	132.90 (19)	C10C—C11C—C12C	120.3 (2)
N1B—C5B—N4B	110.6 (2)	C10C—C11C—H11C	119.8
N1B—C5B—H5B	124.7	C12C—C11C—H11C	119.8
N4B—C5B—H5B	124.7	C13C—C12C—C11C	119.9 (2)
C7B—N6B—N4B	116.38 (19)	C13C—C12C—H12C	120.0
N6B—C7B—C8B	120.4 (2)	C11C—C12C—H12C	120.0
N6B—C7B—H7B	119.8	C12C—C13C—C8C	120.4 (2)
C8B—C7B—H7B	119.8	C12C—C13C—H13C	119.8
C13B—C8B—C9B	119.6 (2)	C8C—C13C—H13C	119.8
C13B—C8B—C7B	118.7 (2)		
			
C5A—N1A—N2A—C3A	0.1 (4)	N6B—C7B—C8B—C13B	176.8 (3)
N1A—N2A—C3A—N4A	0.0 (4)	N6B—C7B—C8B—C9B	−0.7 (4)
N2A—C3A—N4A—C5A	−0.1 (4)	C13B—C8B—C9B—C10B	−1.0 (4)
N2A—C3A—N4A—N6A	−177.9 (3)	C7B—C8B—C9B—C10B	176.5 (3)
N2A—N1A—C5A—N4A	−0.2 (4)	C8B—C9B—C10B—C11B	0.9 (4)
C3A—N4A—C5A—N1A	0.2 (4)	C9B—C10B—C11B—C12B	−0.5 (5)
N6A—N4A—C5A—N1A	178.2 (3)	C10B—C11B—C12B—C13B	0.2 (5)
C5A—N4A—N6A—C7A	−178.2 (3)	C9B—C8B—C13B—C12B	0.7 (4)
C3A—N4A—N6A—C7A	−0.8 (4)	C7B—C8B—C13B—C12B	−176.9 (3)
N4A—N6A—C7A—C8A	178.1 (2)	C11B—C12B—C13B—C8B	−0.3 (4)
N6A—C7A—C8A—C9A	−177.8 (3)	C5C—N1C—N2C—C3C	−0.2 (4)
N6A—C7A—C8A—C13A	1.3 (4)	N1C—N2C—C3C—N4C	0.0 (4)
C13A—C8A—C9A—C10A	−1.2 (4)	N2C—C3C—N4C—C5C	0.2 (4)
C7A—C8A—C9A—C10A	177.9 (3)	N2C—C3C—N4C—N6C	178.9 (3)
C8A—C9A—C10A—C11A	1.3 (5)	N2C—N1C—C5C—N4C	0.3 (4)
C9A—C10A—C11A—C12A	0.0 (5)	C3C—N4C—C5C—N1C	−0.3 (4)
C10A—C11A—C12A—C13A	−1.4 (5)	N6C—N4C—C5C—N1C	−179.2 (3)
C11A—C12A—C13A—C8A	1.4 (4)	C5C—N4C—N6C—C7C	−176.6 (3)
C9A—C8A—C13A—C12A	−0.1 (4)	C3C—N4C—N6C—C7C	4.9 (4)
C7A—C8A—C13A—C12A	−179.2 (3)	N4C—N6C—C7C—C8C	−179.3 (2)
C5B—N1B—N2B—C3B	0.6 (5)	N6C—C7C—C8C—C9C	−177.7 (3)
N1B—N2B—C3B—N4B	−0.7 (5)	N6C—C7C—C8C—C13C	2.7 (4)
N2B—C3B—N4B—C5B	0.4 (4)	C13C—C8C—C9C—C10C	0.3 (4)
N2B—C3B—N4B—N6B	−178.3 (3)	C7C—C8C—C9C—C10C	−179.3 (3)
N2B—N1B—C5B—N4B	−0.4 (4)	C8C—C9C—C10C—C11C	0.2 (4)
C3B—N4B—C5B—N1B	0.0 (4)	C9C—C10C—C11C—C12C	−0.5 (4)
N6B—N4B—C5B—N1B	178.6 (3)	C10C—C11C—C12C—C13C	0.4 (4)
C3B—N4B—N6B—C7B	173.0 (3)	C11C—C12C—C13C—C8C	0.1 (4)
C5B—N4B—N6B—C7B	−5.3 (5)	C9C—C8C—C13C—C12C	−0.4 (4)
N4B—N6B—C7B—C8B	−176.9 (2)	C7C—C8C—C13C—C12C	179.2 (3)

### Hydrogen-bond geometry (Å, °)

**Table:**

*D*—H···*A*	*D*—H	H···*A*	*D*···*A*	*D*—H···*A*
C3C—H3C···N1B	0.93	2.54	3.414 (4)	157
C7C—H7C···N1B	0.93	2.49	3.405 (3)	167
C3A—H3A···N2C^i^	0.93	2.47	3.387 (4)	169
C7A—H7A···N2C^i^	0.93	2.58	3.503 (3)	174
C5B—H5B···N2A^ii^	0.93	2.47	3.371 (4)	162

Symmetry codes: (i) *x*+1/2, *y*+1/2, *z*; (ii) *x*−1/2, *y*−1/2, *z*.
